# Strength and Reaction Time Capabilities of New Zealand Polo Players and Their Association with Polo Playing Handicap

**DOI:** 10.3390/jfmk4030048

**Published:** 2019-07-25

**Authors:** Regan Standing, Russ Best

**Affiliations:** 1 Centre for Sport Science and Human Performance, Wintec, Hamilton 3200, New Zealand; 2 School of Health and Social Care, Teesside University, Middlesbrough TS1 3BX, UK

**Keywords:** Grip Strength, Reaction time, Isometric strength, Polo, Equestrian

## Abstract

Polo is an equestrian team sport consisting of four players per team, with level of play determined by cumulative player handicap (−2 to +10 goals), with a higher handicap denoting a better player. There is minimal literature investigating Polo players’ physical attributes, hence the understanding of the physical characteristics that may contribute to an improved handicap are unknown. This study sought to identify the relationship between pertinent strength measures (left and right hand grip strength; absolute and relative isometric mid-thigh pull) and reaction time in Polo handicap in 19 New Zealand Polo players, and ascertain whether handicap could be predicted by these measures. Correlation coefficients were expressed using R values, accompanying descriptors and 90% confidence intervals (C.I.). Variance explained was expressed via the R^2^ statistic, and statistical significance set at *p* < 0.05. Right hand grip strength, isometric mid-thigh pull values were found to significantly correlate to and explain variance within Polo player handicap (all *moderate* to *large* correlations; *p* < 0.05). Whereas left hand grip strength (R: 0.380; 90% C.I. −0.011 to 0.670) and reaction time (0.020; −0.372 to 0.406) were non-significant, *moderate* and *trivial* correlates and predictors of handicap respectively. Practically, these findings highlight the differing roles between rein and mallet hands of Polo players and emphasise the importance of a strong and stable platform when riding and striking the ball. Lack of association with reaction time may be explained in part by higher handicapped Polo players employing a more proactive approach to the game.

## 1. Introduction

Polo is one of the oldest equestrian sports in the world and requires the synchronisation of both equine and human athletes in a dynamic and high-paced environment [1]. Previous literature has begun to characterise Polo gameplay through global positioning systems (GPS) [2], quantitative performance analyses [3], and equine internal workloads [4,5,6]. These investigations have allowed insight into the science behind Polo and enhanced the discussion of how research may be applied within the sport.

One common factor previous studies have acknowledged, is the subjective handicap rating system used to provide Polo players a quantitative measure of their ability (between −2 and +10) [7]. This system is based on a variety of features including horsemanship, playing skills, technique and the quality of horses being played [7]. Many of the factors contributing to handicap rely on the physical capabilities of the players themselves, however the relationship between physical characteristics and handicap is currently unknown.

Horse riding requires physical strength through both the upper and lower limbs, general cardiovascular endurance, balance, reaction time, and flexibility [8,9,10,11]. In Polo, these physiological attributes may manifest as skilful manipulation of the horse, performance of sudden accelerations or decelerations, and the ability to respond to unpredictable gameplay. Previous equestrian literature has identified kinematic and physiological differences between rider skill levels and equestrian disciplines [8,9,10,12,13], as well as differences in riding skill performance, such as rein tension [9,14], saddle and stirrup pressure [15], and rider asymmetries [9]. This detailed quantification of equestrian elements and their ability to differentiate between performance levels highlights an opportunity for the sports science practitioner in Polo, with testing batteries frequently used in other team sports to this effect [4,16]. The need to identify, train, and evaluate the physical attributes required for effective and safe Polo performance is crucial [17], as players may experience speeds exceeding 60 km/h and distances upwards of 5 km per chukka [1,18] which exposes players to a variety of risks and potential for injury [1].

Current literature has shown greater biomechanical asymmetries within experienced riders than in lesser trained equestrians [9], as Polo is played exclusively right-handed a functional asymmetry in grip strength may be apparent. The stochastic nature and high speeds attained during Polo [19,20] highlight the importance of appropriately forceful and rapid leg actions to communicate with the horse [21]. A strong lower body may also provide a stronger platform for a more rapid segmental rotation and aid control of the mallet through high velocity ball contacts [13]. Reaction times have been shown to be faster in jockeys when compared to track-riders [10]. Despite this trend, due to Polo performance requiring the concomitant integration of multiple actions such as the time it takes for riders to react, the application of pressure to the horse, and the horses corresponding movement, it could be suggested that raw reaction time of Polo players may not be as beneficial to performance as in other sports.

The aim of this study is to quantify the strength and reaction time characteristics of Polo players, and to assess the relationship of these characteristics to player handicap. Findings will provide evidence to inform Polo player training programmes and advise how physical attributes may contribute to improving player handicap. For the reasons outlined above, it is hypothesised that left and right grip strength, and lower limb strength, will possess high correlations to player handicap. It is also hypothesised that reaction time will show little correlation to handicap, as a proactive tactical awareness becomes better developed as experience in the sport increases.

## 2. Materials and Methods

### 2.1. Experimental Approach

Testing consisted of an opportunity sample of Polo players at a licensed New Zealand Polo Association tournament in March 2019 in Cambridge, New Zealand. Testing was conducted pitch-side beyond the requisite safety zone, prior to Polo play. Awareness of this study was raised prior to the tournament through social media posts, with recruitment taking place over the two-day tournament in person. Participants self-reported as being recovered from previous day’s play, which consisted of two four-chukka Polo games. Player handicap was selected as the independent variable, as this is a measure of players’ Polo ability that is awarded by the local Polo governing body (e.g., the New Zealand Polo Association) and reviewed annually; therefore, it could not be manipulated by the researchers. Strength assessments related to horse riding skill or body position (hand grip; isometric mid-thigh pull (IMTP)) and that mimicked the anticipatory requirements of Polo (reaction time) [10,11] were selected as dependent variables. Testing order was at the discretion of participants, details of warm-up and familiarisation procedures are provided below.

### 2.2. Subjects

Nineteen participants (12 male; 7 female) were recruited for this investigation (Handicap: 0 ± 2 goals; Age: 36.2 ± 14.1 y; Weight: 78.9 ± 19.4 kg), all of which had a minimum of two seasons playing experience. Participants’ height was not recorded due to the variability in heel height of players’ Polo boots; performing testing unshod would have breached testing location health and safety regulations due to the close working proximity to horses. Ethical approval for this investigation was awarded by the Waikato Institute of Technology (Wintec) Human Ethics Research Group (Approval code: WTFE02250319), on the 25 March 2019. Participants provided written informed consent prior to undertaking the testing battery and retained the right to withdraw themselves and their data from the study at any time.

### 2.3. Procedures

Left and right-hand grip strength was assessed via a hand grip dynamometer (Smedlay’s, Tokyo, Japan), calibrated up to 100 kg. Grip strength procedures need to mimic the specific demands of the sport to improve the validity of the recording [22]. As such, participants were asked to grip the dynamometer firmly and raise their hand above their head with the palm facing forwards. They were to then squeeze as hard as possible and adduct the shoulder whilst pronating the forearm. The final position was with their arm by their side with the palm facing medially. This protocol was used as it best mimics the dynamics of a Polo swing. Participants self-selected their starting hand but alternated between trials.

Isometric mid-thigh pull (IMTP) was assessed using a customised testing rig, consisting of two Pasco force plates (Roseville, California) and perpendicular vertical poles drilled at 1 cm increments to allow appropriate grip adjustment and positioning of the bar to the participants’ mid-thigh. Intraclass correlation coefficient (ICC) statistics in similar protocols have shown reliable measures both within (ICC = 0.97) and between (ICC = 0.89) sessions [23]. A demonstration of the IMTP was provided by the researcher prior to the first trial, and participants had an opportunity to practise the hand grip placement and pulling position prior to commencement. Participants were provided the opportunity to perform a total of three maximal pulls for this test, with approximately two minutes rest between trials, with the highest net force used for statistical analysis. Peak IMTP net forces were recorded in Newtons (N), and Newtons per kilogram (N/kg) for relative forces, which were calculated by dividing IMTP peak by players’ bodyweights.

Reaction time was assessed via Fitlight reaction lights (Ontario, Canada) set at 30 sec sample duration, with a 0.1 sec delay between lights. Eight lights were mounted on two tables positioned in a right angle and arranged in a fan-like shape around the participant; lights were not placed behind the participants as when mounted on a horse a player cannot leave the confines of the saddle, and to play behind the saddle is considered dangerous. The Fitlight system would randomly activate one of the eight lights that the participant had to wave their hand directly over (approx. 1–3 cm distance from light) to record a single point, this in turn randomly activated another of the eight lights. The total number of lights successfully recorded per 30 s trial was recorded.

Participants were permitted three attempts for each test, following a demonstration by a researcher; participants’ best efforts were used for analysis.

### 2.4. Statistical Analyses

Data were assessed for normality via the Shapiro Wilks test and found to be normally distributed (*p* > 0.05), meaning parametric tests could be employed. Pearson correlation coefficients were used to assess the relationship between Polo handicap and measures of strength and reaction time, with statistical significance set a priori at *p* ≤ 0.05. Ninety percent confidence intervals (C.I.) were used to describe the uncertainty in the data and magnitudes of relationships were described using the following intervals: *Trivial* 0–0.2, *Small* 0.1–0.3, *Moderate* 0.3–0.5, *Large* 0.5–0.7, *Very Large* 0.7–0.9 and *Nearly Perfect* >0.9 [24]. Variance explained was expressed via the R^2^ statistic.

Linear regression was used to determine the predictive ability of Polo handicap upon strength variables and reaction time, with relationships described using the formula *y* = a + b*x*; where y is the dependent variable score, a is the intercept on the y axis, b is the slope of the regression line and x is the Polo handicap. A paired samples t-test was used to assess statistical differences between left and right grip strengths; this comparison is presented with a *p* value, Hedge’s *g,* 90% C.I., and qualitative description of magnitude. For clarity, correlation coefficients, *p* values, and R^2^ values are stated to three decimal places. All data analysis was conducted in SPSS (IBM SPSS Statistics version 24, IBM, location); confidence intervals for correlation coefficients were calculated using a customised spreadsheet [25].

## 3. Results

Group means identified handgrip strength was greater in the right hand (50.9 ± 16.6 kg) when compared to the left (46.3 ± 15 kg). As depicted in Table 1, both left and right handgrip strengths displayed Moderate to Large correlations to player handicap, with significance achieved by the right hand only (*p* = 0.019). Right hand grip strength was significantly different (*p* = 0.019) to left hand grip strength (Hedges *g*: 0.275, 90% C.I.: 0.086 to 0.490; *Small*).

Mean values for IMTP and mid-thigh isometric pull relative to player bodyweight (IMTP-R) were 1888.3 ± 597.2 N and 23.9 ± 5.52 N/kg, respectively. Significant relationships to player handicap were also demonstrated by IMTP (*p* = 0.004) and IMTP-R (*p* = 0.035), which displayed correlations to player handicap of 0.609 and 0.484, respectively. Reaction time was shown to have a non-significant relationship (*p* = 0.889) to player handicap, with a group mean of 23.3 ± 2.7 light responses per 30 s testing window.

**Figure 1:**
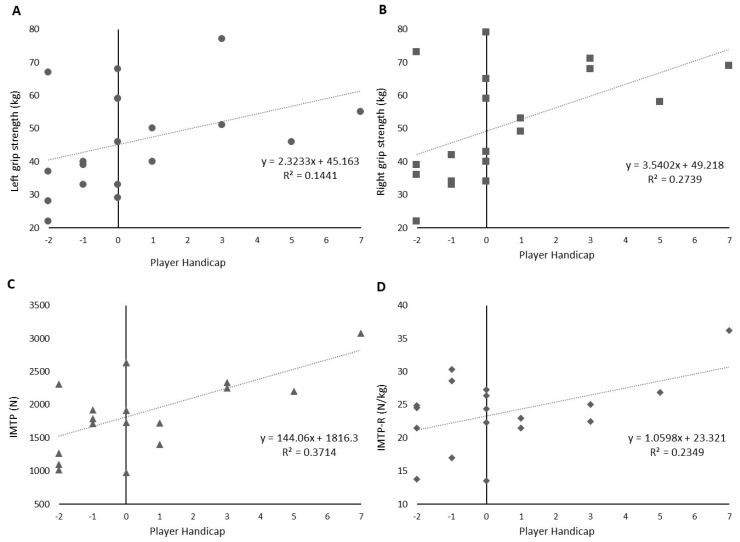
Individual data points and accompanying linear regression lines for left (**A**) and right (**B**) hand grip strength, IMTP (**C**) and IMTP-R (**D**).

All variables that displayed significant relationships to handicap (right handgrip strength, IMTP and IMTP-R) also demonstrated significant R^2^ values, suggesting that these metrics may be predictive of Polo handicap. Regression equations for each variable can be found in Table 1; individual data plots for each variable that displayed moderate to large relationships with player handicap, with accompanying regression lines are depicted in Figure 1A–D.

## 4. Discussion

The purpose of this study was to characterise strength and reaction time attributes of Polo players and assess the relationship between these factors and player handicap. This study shows that right-hand grip strength, IMTP, and IMTP-R have significant relationships to player handicap. However, reaction time neither correlates to nor is predictive of player handicap, therefore supporting the hypothesis of this paper. Left-hand grip strength presented a non-significant *moderate* relationship with player handicap, which was contrary to the initial hypothesis.

Previous literature has highlighted the range of handgrip strength values that are present across various sporting codes [22], with dressage horse riders displaying some of the lowest hand grip values (<30 kg) [26] and rowers displaying some of the highest (>70 kg) [27]. In the current study, handgrip strength was higher than previous equine-based investigations [18,26,28], although the methods of collecting handgrip strength differed based on the event specific requirements of the various equestrian pursuits examined. Differences in hand grip testing methodology have also been shown to influence the validity attainment of maximal measures in some instances and therefore, this comparison of hand grip data between studies should be interpreted cautiously, as testing procedures differed between most studies in an attempt to employ a valid result for each sporting context may account for some of the variation identified [22]. It is suggested that handgrip demands differ between equestrian events, and with the added intensity, speed and manoeuvrability required in Polo, a stronger grip may in fact be more advantageous. With the added need to manipulate the mallet with the right-hand, strength becomes important to repeatedly control impacts on the ball and produce consistent shots. Non-significant correlations (0.380; 90% C.I. −0.011 to 0.670 *p* = 0.102) and decreased grip strength were observed in the left hand. This may be explained by the riding style required for Polo [29], where finesse and intricate controlled movements are used to manoeuvre the horse via the left-hand on the reins, and not necessarily through strong and forceful movements as initially hypothesised. The left to right asymmetry may also be described by the right-hand dominance which is witnessed in 80–90% of demographic studies [22,30,31]. Whilst using one hand to swing the mallet, and the other to manipulate the horse, the need to remain stable in the saddle is also of critical importance.

In jockeys, leg strength and power are more positively associated with falls than in track-work riders [10], despite better balance scores. This may be a product of work-related exposure to more fractious horses [10]; it is not unreasonable to suggest similar trends may be apparent in Polo, as horsemanship is a requisite of increased handicap [7]. Stability in the saddle is determined by the interaction of various factors, namely the horse, type of saddle, rider and the type of movements being performed [32]. Stability is maintained by the rider’s ability to follow the movements of the horse and by using both legs to provide the base for this movement [32,33]. IMTP-R and IMTP displayed *moderate* to *large* relationships with player handicap and significant R^2^ values of 0.235 and 0.371, respectively, highlighting the predictive qualities of these measures, with respect to player handicap. Mean values for IMTP and IMTP-R in Polo players are comparable to those of recreationally strength trained males [34], but ~10% less than professional soccer players [35] and ~20% lower than that of elite male surfers [36]. To date, IMTP values for other equestrian populations have not been published.

There is a clear need for a strong base of support, and the ability to produce high levels of force on the stirrups through both legs whilst Polo players are riding at speed, playing shots out of their saddle [13] and absorbing contacts from different angles (ride-off; [20]). Estimated stirrup forces at a rising trot are 2.34 times rider bodyweight [15], the forces exerted during higher velocity gaits are currently unknown. To date, no studies have assessed the plantar pressure exerted on the stirrups during Polo specific movements. However, evidence from general equestrian literature highlights the role of downward pressure on the stirrups and leg pressure around the horse to decelerate, turn and effectively communicate rider intent [21]. Proper foot positioning further serves to minimize risk of dismount, entrapment and injury to the rider [37]. More experienced riders demonstrate dorsiflexion at the ankle in comparison to the plantar flexed position of novice riders [9,38] and are better able to attenuate the increased vertical forces imparted by increasing horse gait speeds [9]. These findings suggest a strong platform at the foot and ankle and throughout the lower limb is likely advantageous and a learned product of riding experience. This may partially explain the large association between IMTP and handicap in the present study. Furthermore, at faster riding speeds riders likely adopt a two-point seat (stood on stirrups), which has been noted to be metabolically expensive for the rider [9], but energy saving for the horse [39].

There is a paucity of literature surrounding lower limb strength in horse riders, therefore the need to discuss the relationship between lower limb strength and handicap warrants further investigation within the Polo context. The methods utilised in the current study using the IMTP provide a typically static, yet reliable measure of outright lower limb strength, but the oscillatory nature of riding and stochastic nature of Polo presents a unique opportunity for future research to explore various methods of measuring this strength in a Polo specific manner. The use of the legs as opposed to the reins (left hand grip strength) to decelerate and turn the horse will preserve the integrity of horses’ mouths and tongues, which are prone to oral injury through Polo participation [40]. Indeed, light rein contact is preferred across equestrian disciplines and is considered to yield better equine responses [12], but it is acknowledged that each horse-rider interaction is highly variable [14,41] and the application of force through the lower limb or the rein hand will impose differing biomechanical constraints upon both the horse and rider.

Reaction time data showed a *trivial* non-significant relationship to handicap, which is dissimilar to previous literature pertaining to reaction time in equestrian pursuits, and sports requiring high speed and agility characteristics [42,43]. It is hypothesized that better Polo players may employ a proactive strategy and predict plays, potentially limiting the requirement for fast reaction times. In response to gameplay, interactions between the rider and the horse ultimately create an action based on the rider’s perception of what is required. As handicap increases players likely foster an ability to read the game, respond more efficiently and how to manipulate their horses accordingly. These skills are contributors to ‘horsemanship’ and ‘playing skills’, two of the categories considered when player handicap is awarded [7]. It is important to note, that the physical characteristics measured within this study are not directly measured to influence or attain player handicap ratings. These variables do however contribute to the players’ ability to perform the subjectively measured aspects related to Polo play.

### Practical Applications

Without consistent and objective handicap profiling procedures, it is difficult to make conclusive statements about how players may be able to utilise these findings to improve their handicap. However, results of this study suggest practitioners working with Polo players, or other equestrian pursuits, should focus on the development of grip strength, as well as the riders’ ability to transfer force through their lower limb as this provides a stronger platform on the stirrup when playing on-ball. Time spent developing players’ ability to read the game and make proactive moves may be a more effective use of time than training reactive components. Future research should further investigate the bilateral differences between left and right hands of Polo players, and the motor nuance required to perform most effectively. Lower limb strength and endurance capacities should also be investigated within Polo, and could be used in conjunction with player heart rates to clarify central or peripheral limitations [44]. Further information pertaining to the internal physical demands and external workloads of Polo would further aid in training programmes for Polo players.

## Figures and Tables

**Table 1:** Correlation coefficients between Polo handicap and strength and reaction time (RT). Accompanying 90% Confidence intervals (C.I.), p values and magnitude descriptors are also shown. Variance explained (R^2^) and the linear regression equations are also presented for each variable, as per Polo handicap. HG: Handgrip; IMTP: Isometric mid-thigh pull; IMTP-R: Isometric mid-thigh pull relative to bodyweight; RT: Reaction time: Significant values (*p* < 0.05) are denoted by an asterisk *.

Variable	Correlation	90% C.I.			*p* Value	Descriptor	R^2^ Value	Regression Equation
HG Left	0.380	−0.011	to	0.670	0.102	*Moderate*	0.144	*y* = 2.387*x* + 44.362
HG Right	0.523	0.168	to	0.758	0.019 *	*Large*	0.274 *	*y* = 3.613*x* + 48.305
IMTP	0.609	0.275	to	0.812	0.004 *	*Large*	0.371 *	*y* = 148.030*x* + 1766.396
IMTP-R	0.484	0.103	to	0.741	0.035 *	*Moderate*	0.235 *	*y* = 1.065*x* + 23.258
RT	0.020	−0.372	to	0.406	0.889	*Trivial*	0.001	*y* = −0.037*x* + 23.463
